# Halophyte *Nitraria billardieri CIPK25* mitigates salinity-induced cell damage by alleviating H_2_O_2_ accumulation

**DOI:** 10.3389/fpls.2022.961651

**Published:** 2022-08-08

**Authors:** Lu Lu, Xinru Wu, Pengkai Wang, Liming Zhu, Yuxin Liu, Yao Tang, Zhaodong Hao, Ye Lu, Jingbo Zhang, Jisen Shi, Tielong Cheng, Jinhui Chen

**Affiliations:** ^1^Key Laboratory of Forest Genetics & Biotechnology of Ministry of Education of China, Co-Innovation Center for Sustainable Forestry in Southern China, Nanjing Forestry University, Nanjing, China; ^2^Experimental Center of Desert Forestry, Chinese Academy of Forestry, Dengkou, China; ^3^College of Biology and the Environment, Nanjing Forestry University, Nanjing, China

**Keywords:** halophyte, *Nitraria billardieri*, *CIPK25*, salt tolerance, H_2_O_2_

## Abstract

The plant-specific module of calcineurin B-like proteins (CBLs) and CBL-interacting protein kinases (CIPKs) play a crucial role in plant adaptation to different biotic and abiotic stresses in various plant species. Despite the importance of the CBL-CIPK module in regulating plant salt tolerance, few halophyte CIPK orthologs have been studied. We identified *NbCIPK25* in the halophyte *Nitraria billardieri* as a salt-responsive gene that may improve salt tolerance in glycophytes. Sequence analyses indicated that *NbCIPK25* is a typical CIPK family member with a conserved NAF motif, which contains the amino acids: asparagine, alanine, and phenylalanine. *NbCIPK25* overexpression in salt-stressed transgenic *Arabidopsis* seedlings resulted in enhanced tolerance to salinity, a higher survival rate, longer newly grown roots, more root meristem cells, and less damaged root cells in comparison to wild-type (WT) plants. H_2_O_2_ accumulation and malondialdehyde (MDA) content were both deceased in *NbCIPK25*-transgenic plants under salt treatment. Furthermore, their proline content, an important factor for scavenging reactive oxygen species, accumulated at a significantly higher level. In concordance, the transcription of genes related to proline accumulation was positively regulated in transgenic plants under salt condition. Finally, we observed a stronger auxin response in salt-treated transgenic roots. These results provide evidence for *NbCIPK25* improving salt tolerance by mediating scavenging of reactive oxygen species, thereby protecting cells from oxidation and maintaining plant development under salt stress. These findings suggest the potential application of salt-responsive *NbCIPK25* for cultivating glycophytes with a higher salt tolerance through genetic engineering.

## Introduction

Some plant species have evolved multifaceted mechanisms to mitigate adverse stress effects and improve their tolerance toward extreme environmental conditions such as salt stress (Zhu, 2002). Soil salinity is one of the major limiting conditions for plant growth, inducing both osmotic and toxicity stress in plants, subsequently leading to growth and developmental inhibition (van Zelm et al., 2020). One type of toxicity stress that salt may induce is oxidative stress, which is involved in the homeostasis of the reactive oxygen species (ROS). ROS are important signaling molecules that have been revealed to affect plant response to environmental stresses, regulating programmed cell death (PCD) and hormone signaling (Xie et al., 2014; You et al., 2014; Leng et al., 2017; Tognetti et al., 2017; Zhang et al., 2022). ROS signaling disruption causes defects during plant development (Guillou et al., 2022; Zhang et al., 2022), activating detoxification pathways for remediation of salinity-induced damage (Zhu, 2002). Therefore, for adaptive responses, plants have evolved specific mechanisms to regulate the production and scavenging of ROS through enzymatic and non-enzymatic antioxidative processes (Nadarajah, 2020; Dias et al., 2022; Šoln and Koce, 2022).

Another molecule that is involved in plant response to abiotic stresses is proline (Szabados and Savouré, 2010; Kavi Kishor and Sreenivasulu, 2014). Proline synthesis is catalyzed by the pyrroline-5-carboxylate synthetase (P5CS) and P5C reductase (P5CR) (Hu et al., 1992). P5CS is rate-limiting for proline synthesis and has two isoforms (P5CS1 and P5CS2), and each has its function in *Arabidopsis*. P5CS1 mediates stress-induced proline accumulation, while P5CS2 acts as a housekeeping enzyme (Funck et al., 2012; Mattioli et al., 2018; Funck et al., 2020). Proline degradation is regulated by proline dehydrogenase (PDH) (Szabados and Savouré, 2010). Numerous studies have demonstrated that proline accumulation is induced by cold, osmotic, and salt stresses (Kavi Kishor and Sreenivasulu, 2014; Hoermiller et al., 2022; Yan et al., 2022). Proline has been found to improve stress tolerance in many plants including rice, chili, and Zea mays through scavenging ROS (Xiang et al., 2007; Abdula et al., 2016; Bhusan et al., 2016; Alam et al., 2017; Hosseinifard et al., 2022). For example, exogenous proline application reduces Hg^2+^ toxicity in rice, while ROS content was alleviated by P5CS overexpression in tobacco plants (Hong et al., 2000; Siripornadulsil et al., 2002; Wang et al., 2009). In addition, in the *Arabidopsis p5cs1* mutant, a reduction in proline synthesis leads to ROS accumulation, which in turn elevates oxidative stress (Szekely et al., 2007).

Among the known salt-responsive pathways, the calcineurin B-like protein (CBL) and CBL-interacting protein kinase (CIPK) signaling module has been well studied (Yin et al., 2019; Tang et al., 2020). CBLs perceive changes in Ca^2+^ signaling and alter their conformation to specifically bind CIPKs. The activated CIPKs thus phosphorylate their downstream target proteins such as ion channels, phosphatases, transporters, and transcription factors (Sheng et al., 2009; Tang et al., 2015; Straub et al., 2017; Reyes and Grégory, 2021). The CIPK family genes encode a unique C-terminal regulatory region with a conserved NAF (Asn-Ala-Phe) motif that is required for interaction with CBLs (Albrecht et al., 2001). Besides, CIPKs possess a highly conserved N-terminal kinase domain that facilitates the phosphorylation of proteins involved in adaptation to multiple stresses (Kudla et al., 1999; Albrecht et al., 2001; Kim, 2003).

During salt stress, *Arabidopsis* CIPK24 (also known as SOS2), a crucial member of the salt overly sensitive (SOS) pathway, alleviates salt stress by activating SOS1 to stimulate Na^+^/H^+^ exchange across the plasma membrane (Liu et al., 2000; Guo et al., 2001; Yin et al., 2019). In addition, *Arabidopsis* CIPK8 has also been validated to target SOS1 for Na^+^/H^+^ exchange under salt stress (Yin et al., 2019). Another *Arabidopsis* CIPK protein, AtCIPK6, increases plant tolerance to salt stress when overexpressed in *Arabidopsis* (Chen et al., 2013). In other plant species, an increasing number of CIPK family members were recognized as salt-tolerance factors. Maize *ZmCIPK42* overexpression enhanced salt tolerance, while plants with a mutation in the same gene showed impaired salt stress tolerance (Chen et al., 2021). Glycine *GmCIPK21* was implicated in affecting ABA response and ROS homeostasis to improve the salt tolerance of soybean (Li et al., 2022). A CIPK gene from chickpea, *CaCIPK6*, has been shown to mediate auxin transport to regulate the salt tolerance of tobacco seedlings (Tripathi et al., 2010). Apple and Poplar *CIPK24*s improve salt tolerance by increasing the proline content and the activities of antioxidant enzymes to balance osmotic pressure and mitigate salinity-induced damage (Zhou et al., 2014; Hu et al., 2015). Taken together, these studies have demonstrated the pluralistic mechanisms through which *CIPK* function can improve salt tolerance in various plant species.

To elucidate the mechanisms underlying a plant’s response to growing in saline soil, to date mostly glycophytes have been studied. Unlike salinity-tolerant halophyte plants, however, glycophytes have relatively poor tolerance to salt stress, and thus only a few functional genes can be utilized to improve salt tolerance (Flowers and Colmer, 2010; Cheeseman, 2014). *Nitraria billardieri* (*N. billardieri*) is a typical halophyte that grows in arid deserts and saline grasslands and is routinely planted to assist in stabilizing sand deposits and reducing soil salinity (Zhang et al., 2015). Thus, *Nitraria* species have unique physiological characteristics that are relevant to growth in saline soils and are thus ideal for studying the mechanisms of salt tolerance (Li et al., 2021). Therefore, we utilized *N. billardieri* to identify the gene involved in tolerance to salt and drought. The *NbCIPK25* gene was found to positively respond to salt and drought stress in *N. billardieri*. Expression of *NbCIPK25* in *Arabidopsis* enhances salt tolerance and maintains the root development of transgenic plants. Furthermore, *NbCIPK25* transgenic roots suffer less H_2_O_2_ accumulation and cell damage, showing that *NbCIPK25* protects cells from ROS-induced damage in salt-stressed plants. These results indicate that *NbCIPK25* has a dominant role in plant salt tolerance, suggesting that its transgenic expression may provide a practical means of increasing the salt tolerance of other glycophyte species.

## Materials and methods

### Plant materials and treatments

*N. billardieri* seeds were kindly provided by the Experimental Center for Desert Forestry of the Chinese Academy of Forestry. One-month-old seedlings of *N. billardieri* were irrigated with water containing 500 mM NaCl or 200 mM mannitol. Leaves, stems, and roots were then harvested after a 2 h stress treatment for RNA extraction. Each treatment included three biological replicates for each of three independent experiments.

The *Arabidopsis thaliana* (*A. thaliana*) Columbia ecotype was utilized to produce *NbCIPK25* transgenic plants. *NbCIPK25* homozygous transgenic seeds were used for phenotype observation and physiological analysis. To analyze the function of *NbCIPK25* on salt tolerance, WT and transgenic seeds were sown on ½ Murashige-Skoog (½MS) medium until germination. Then, 5-day-old seedlings were transferred onto a medium containing 0 mM, 100 mM, and 150 mM NaCl, respectively. After 3 days, root length and the degree of chlorosis were determined. A *t*-test was used for statistical analysis.

To assess the effect of *NbCIPK25* on root meristem development, homozygous *NbCIPK25* transgenic *Arabidopsis* plants were crossed with C24 accession *Arabidopsis* plants containing J2341:GFP fluorescence, which functions as a marker for root columella initial cells (Pi et al., 2015). To analyze auxin distribution at the root meristem, *NbCIPK25* transgenic plants were crossed with plants containing the DR5:GFP transgene.

### *NbCIPK25* cloning and sequence analysis

Total RNA was extracted from the leaves of *N. billardieri* seedlings using the Eastep Super Total RNA Purification Kit (Promega, Shanghai, China), followed by the removal of genomic DNA contamination using DNase I contained in the kit. cDNA was synthesized according to the manufacturer’s instructions using the HiScript III 1st Strand cDNA Synthesis Kit (+gDNA wiper) (Vazyme Biotech, Nanjing, China). Specific primers for *NbCIPK25* isolation (Supplementary Table 1) were designed based on the predicted sequence of *NbCIPK25* from the unpublished transcriptome data of *N. billardieri* in our lab. The 5′ and 3′ untranslated region of *NbCIPK25* was verified with 5′ and 3′ Rapid amplification of cDNA ends (RACE) using the primers listed in Supplementary Table 2 following the SMARTerTM RACE cDNA Amplification Kit User manual (BD Bioscience Clontech, United States). The complete coding sequence of *NbCIPK25* was confirmed from cDNA based on the assembled RACE sequences using the primers mentioned in Supplementary Table 3.

Multiple amino-acid sequence alignment of NbCIPK25 and its orthologs were achieved using DNAMAN 6.0. Feature motifs and conserved domains in NbCIPK25 were analyzed with InterProScan online software^1^. Accession numbers for sequences of all CIPK25 orthologs used for the multiple alignments are listed in Supplementary Table 4. The prediction of hydrophobic sequences and transmembrane domains for NbCIPK25 was performed using ProtScale^2^ and TMHMM Server 2.0^3^, respectively. Evolutionary studies of NbCIPK25 and 26 CIPKs from *Arabidopsis* or CIPK25 orthologs from various plant species were performed with amino-acid sequences downloaded from NCBI using Mega 6 in the neighbor-joining method with 1,000 bootstrap replications and the JTT model. The analysis for conserved motif distribution of CIPK25 proteins was performed by the MEME website with 10 as the maximum motif number^4^ (Marchler-Bauer et al., 2015). Accession numbers for CIPK sequences used for the phylogenetic analysis are listed in Supplementary Tables 5, 6.

### Quantitative real-time PCR analyses

To confirm the response of *NbCIPK25* to salt and drought stress, quantitative real-time PCR (qPCR) analysis was performed using total RNA extracted from the root, stem, and leaf tissues of 1-month-old *Nitraria* plants treated with 500 mM NaCl or 200 mM mannitol for 2 h. Tissue samples from 5-day-old *NbCIPK25*-overexpressing *Arabidopsis* and WT plants treated with 100 mM NaCl were harvested to analyze the transcription of genes involved in proline metabolism. Total RNA was reversely transcribed as mentioned previously.

qPCR was carried out using TB Green^®^ Premix Ex Taq™ (Takara, Dalian, China) with a LightCycler^®^480 qPCR detection system (Roche, Basel, Switzerland). The expression of target genes was normalized by the expression level of the housekeeping gene actin in *Nitraria* (Wang et al., 2012) and *UBQ10* in *Arabidopsis* (Norris et al., 1993). Three biological and experimental repeats were performed. The accession numbers of genes tested with qPCR and the primers used are listed in Supplementary Table 7.

### Tissue staining, microscopy, and image analysis

Trypan blue staining of cells was carried out as reported previously (Qiao et al., 2010). The in-situ accumulation of H_2_O_2_ and O_2_^–^ was visualized via staining with 3,3’-diaminobenzidine (DAB) and nitrotetrazolium blue chloride (NBT), respectively, as described previously (Romero-Puertas et al., 2010). Seedlings were stained with 10 μM propidium iodide (PI) (Sigma) for 30 min to quantify the number of cells with a damaged membrane.

The DAB, NBT, and trypan blue staining were imaged with a Leica M165FC microscope. Roots expressing GFP were prepared and mounted in 10 μM PI for 30 min before imaging. Confocal images were captured with LSM software (ZEN blue edition 2.6 system; Zeiss, Germany) on a laser scanning microscope (LSM 800 system; Zeiss). Gray level analyses for images were calculated with ImageJ software.

### Proline, H_2_O_2_, and malondialdehyde content measurement

Proline, H_2_O_2_, and malondialdehyde (MDA) were quantified with kits purchased from Sangon Biotech (D799576, D799774-0100, and D799762-0100, Shanghai, China) following the user manual for each kit.

### Data processing

A student’s *t*-test was carried out using GraphPad Prism v.8 for all the statistical analyses to determine the significance of phenotypical differences observed between WT and transgenic plants undergoing identical treatments (*p < 0.05; **p < 0.01; ***p < 0.001; n.s., not significant).

## Results

### *NbCIPK25* identification and bioinformatic analysis

**FIGURE 1:**
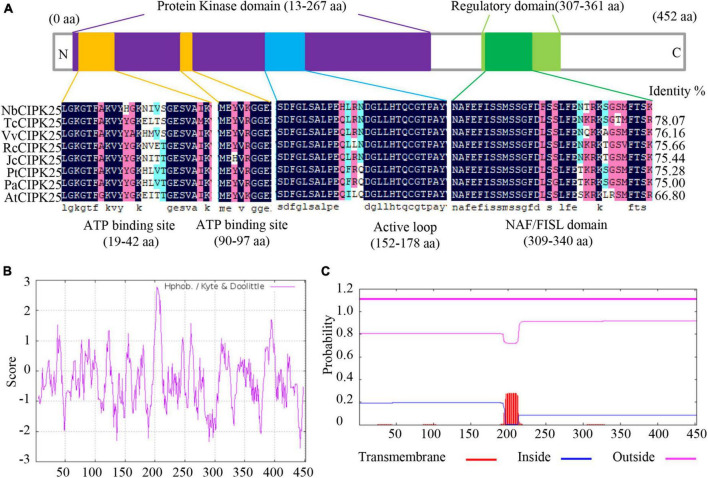
*NbCIPK25* identification by sequence analysis. **(A)** The conserved domain of NbCIPK25 compared with homologous genes from other species, including *Theobroma cacao* CIPK25 (TcCIPK25), *Vitis vinifera* CIPK25 (VvCIPK25), *Ricinus communis* CIPK25 (RcCIPK25), *Jatropha curcas* CIPK25 (JcCIPK25), *Populus trichocarpa* CIPK25 (PtCIPK25), *Populus alba* CIPK25 (PaCIPK25), and *Arabidopsis thaliana* CIPK25 (AtCIPK25). **(B)** Hydrophobicity plot of NbCIPK25. **(C)** The predicted transmembrane domain of NbCIPK25.

Halophytes have been considered a potentially crucial resource of salt-tolerant genes due to their strong resistance to salinity. To explore the utility of such genes, we cloned a previously unidentified CIPK from the halophyte *N. billardieri*. The alignment of CIPK homologs revealed a high similarity between the deduced amino-acid sequence of the newly cloned *N. billardieri* gene and CIPK25s from various plant species (Figure 1A). Like other CIPKs, the protein sequence of the obtained gene contains an N-terminal SNF-1-related serine/threonine protein kinase domain and a C-terminal regulatory domain with a CBL-interacting NAF/FISL module (Figure 1A). Analysis of the hydrophobicity using ProtScale revealed residues 198 to 210 of the obtained protein as the most hydrophobic sequence (Figure 1B). The same protein sequence was also predicted to interact with the cellular membranes based on analyses performed with TMHMM Server 2.0, which indeed predicted the most hydrophobic sequence to be a membrane-binding site (Figures 1B,C).

**FIGURE 2:**
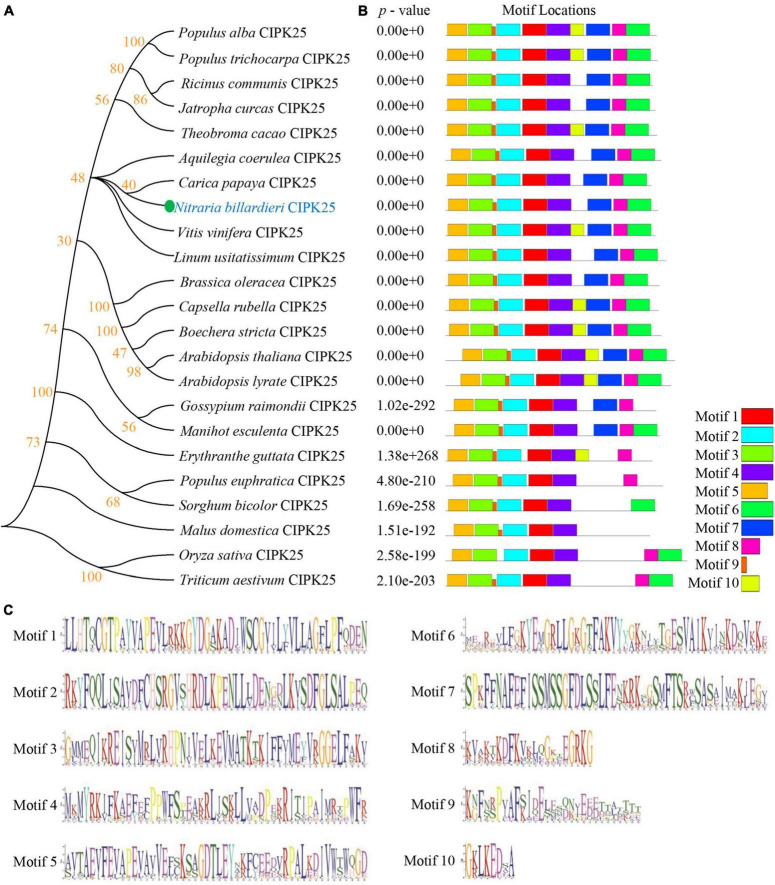
Phylogenetic study and conserved motif analysis of CIPK25s. **(A)** The phylogenetic relationship between NbCIPK25 and 22 CIPK25 from indicating species. The tree was made using the Neighbor-Joining method in Mega 6 with 1,000 bootstraps. **(B)** The conserved motif distribution of CIPK25 proteins. The 10 boxes indicate conserved motifs with different colors. **(C)** Conserved sequences of the 10 motifs identified in CIPK25 proteins from Figure 2B.

**FIGURE 3:**
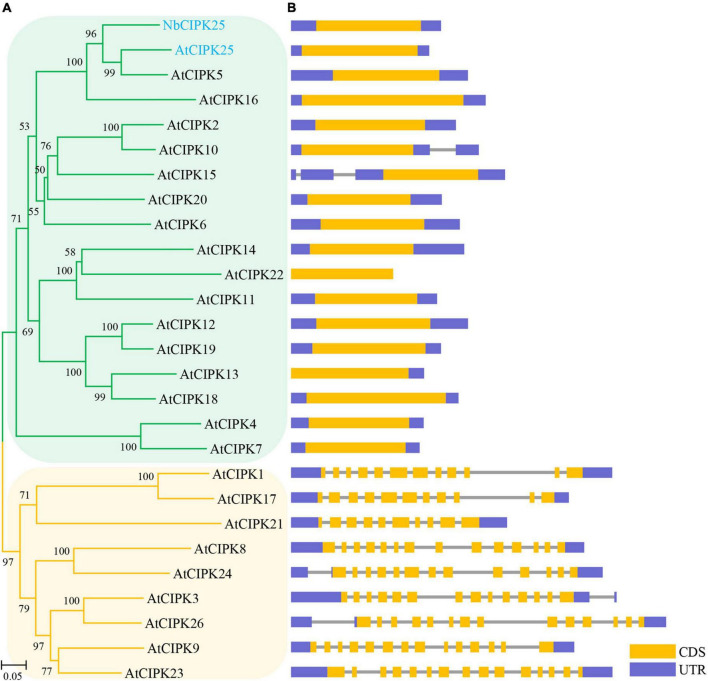
Evolutionary analysis of NbCIPK25 and *Arabidopsis* CIPKs. **(A)** 26 CIPKs from *Arabidopsis* and NbCIPK25 were used for a phylogenetic study; The genes clustering in the orange branches contain introns in their genomic sequences. The intron-less genes are clustered in the green branches. **(B)** Architectures of *NbCIPK25* and *AtCIPK*s including their coding sequence (CDS) and untranslated region (UTR).

Our phylogenetic analyses showed that the protein encoded by the newly cloned gene and *Vitis vinifera* (*V. vinifera*) CIPK25 resides on the same branch of the phylogenetic tree (Figure 2A). The distribution of conserved motifs also showed a strong identity between the protein sequence of the obtained gene with CIPK25s from other species (Figures 2B,C). Furthermore, a phylogenic study of 27 CIPKs revealed that the new CIPK from *N. billardieri* clustered into a sister branch with *A. thaliana* AtCIPK25 in the intron-less clade (Yu et al., 2007; Figures 3A,B). Hence, we designated the newly cloned gene as *NbCIPK25*, a novel member of the halophyte *CIPK* family.

### Stress-induced tissue-specific expression of *NbCIPK25* in *Nitraria billardieri*

**FIGURE 4:**
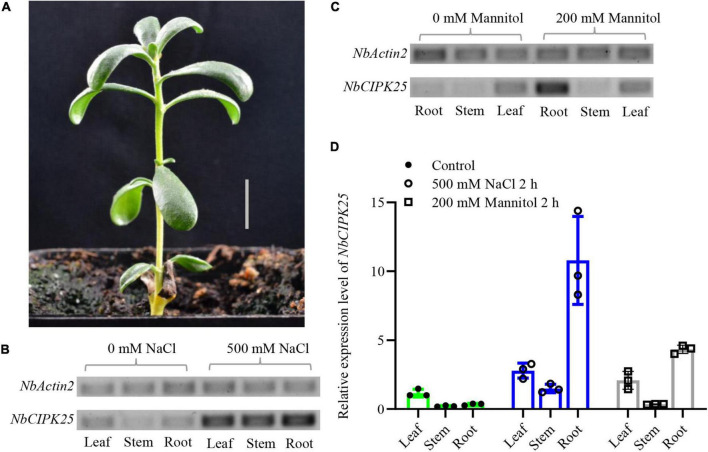
Stress-induced *NbCIPK25* expression in different tissue of *N. billardieri*. **(A)** Morphology of *N. billardieri* seedlings used for transcription level analysis of *NbCIPK25* under salt and drought treatment. Scale bar: 1 cm. **(B,C)** *NbCIPK25* expression was tested by semi-qPCR in the indicated tissues of *N. billardieri* exposed to 500 mM NaCl **(B)** and 200 mM mannitol for 2 h **(C)**. **(D)** *NbCIPK25* expression tested by qPCR in the indicated tissues of *N. billardieri* exposed to 500 mM NaCl and 200 mM mannitol for 2 h. Data represent the means ± SD, three biological replicates.

To assess whether *NbCIPK25* responds to salt stress in *N. billardieri*, total mRNA was isolated from root, leaf, and stem tissues to quantify transcript abundance. Results from a semi-quantitative PCR (semi-qPCR) analysis showed the highest *NbCIPK25* expression in leaf tissue from plants under normal conditions. Treating *N. billardieri* plants with 500 mM NaCl for 2 h, however, led to upregulated expression of *NbCIPK25* in leaf, stem, and particularly root tissue (Figures 4A,B). Furthermore, qPCR data for the expression profiling of *NbCIPK25* yielded similar results. The data showed that 500 mM NaCl treatment caused upregulation of *NbCIPK25* expression by 2. 7-, 1. 4-, and 10.8-fold in leaf, stem, and root tissue, respectively, compared with the expression in leaves of untreated plants (Figure 4D). A similar, yet weaker trend of tissue-specific expression was observed when plants were treated with drought stress simulated by applying 200 mM mannitol (Figures 4C,D). These results show that *NbCIPK25* is upregulated in response to salt and drought stresses, especially in the root.

### *NbCIPK25* mitigates the negative effects of salt stress on *Arabidopsis* development

**FIGURE 5:**
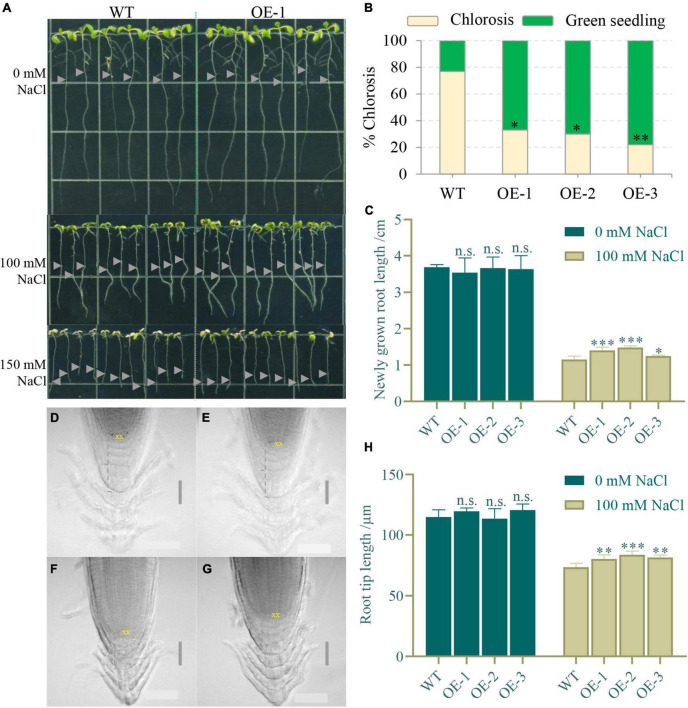
*NbCIPK25* overexpression maintains root development under salt stress. **(A)** The 5-day-old WT and *NtCIPK25* overexpressing plants (OE-1, OE-2, and OE-3 representing three individual lines) grew on a medium containing 0 mM NaCl, 100 mM NaCl, and 150 mM NaCl for 3 days; gray triangles indicate starting points of root tips on the respective medium per treatment. Square length: 1 cm. **(B)** Percentage of chlorosis for WT and transgenic seedlings treated with 150 mM NaCl for 3 days. **(C)** Newly grown length of the primary root. **(D–G)** Micrographs showing the primary root tip of WT **(D)** and transgenic seedlings **(E)** treated with 0 mM NaCl for 3 days; and the primary root tip of WT **(F)** and transgenic seedling **(G)** treated with 100 mM NaCl for 3 days; scale bar: 50 μm. “XX” indicates the quiescent center (QC). **(H)** The root tip length is measured along the dashed line indicated in **D–G**. **(B,C,H)** Data represent means ± SD, three biological replicates, and a t-test was used to determine statistical significance, “*” *p* < 0.05, “**” *p* < 0.01, “***” *p* < 0.001, n.s.: not significant.

To study whether *NbCIPK25* expression affects plant salt tolerance, we constructed transgenic *Arabidopsis* plants overexpressing *NbCIPK25*, using only homozygous seeds for further study. The 5-day-old seedlings germinating under normal conditions were transferred to a medium containing 0, 100, or 150 mM NaCl. We observed no significant growth difference between WT and transgenic plants in the absence of NaCl (Figure 5A). However, when grown in a medium containing 150 mM NaCl for 3 days, transgenic plants showed a significantly lower incidence of chlorosis than in WT (Figure 5B). Moreover, newly grown roots of transgenic seedlings were longer than those of WT seedlings on 100 mM NaCl, suggesting more root growth activity (Figures 5A,C). In addition, the root tip length under the quiescent center (QC) of transgenic plants was observed to be significantly greater than that of WT plants under salt treatment (Figures 5F–H), although there was no significant difference under normal conditions (Figures 5D,E,H). This phenotype implies that *NbCIPK25* maintains root development in the presence of salt.

**FIGURE 6:**
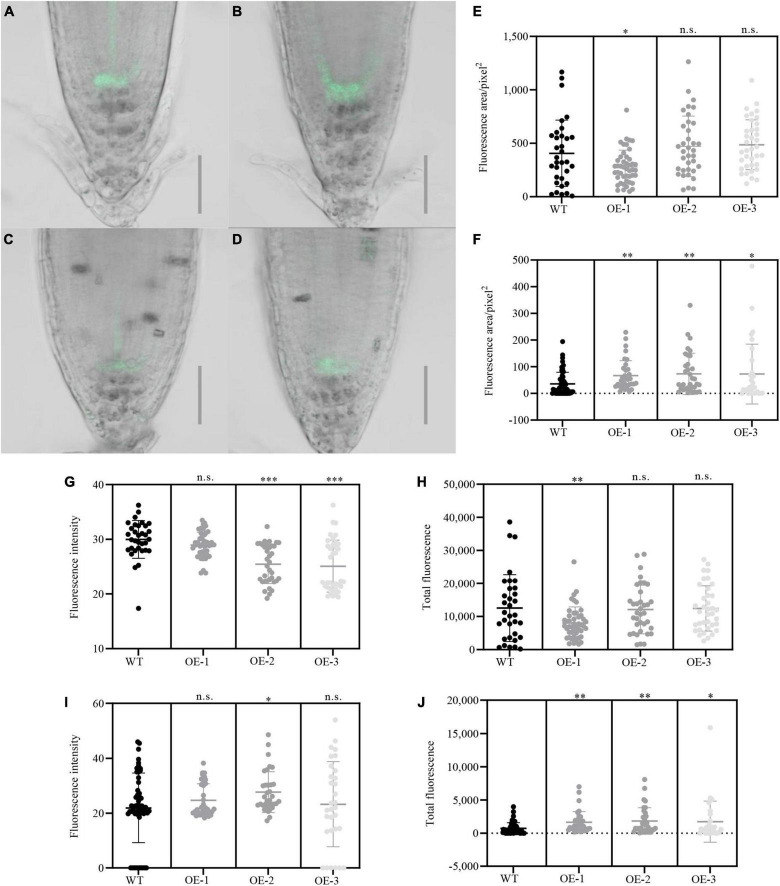
*NbCIPK25* affects the activity of root meristem cells under salt stress. **(A–D)** Green fluorescence indicates the J2341:GFP signal in WT **(A)** and transgenic seedlings **(B)** treated with 0 mM NaCl for 24 h; J2341:GFP in WT **(C)**, and transgenic seedlings **(D)** treated with 100 mM NaCl for 24 h; scale bar: 50 μm. **(E–J)** Fluorescence area **(E)**, fluorescence intensity **(G)**, and total fluorescence **(H)** of the J2341:GFP signal in the 5-day-old WT and transgenic seedlings treated with 0 mM NaCl for 24 h; Fluorescence area **(F)**, fluorescence intensity **(I)**, and total fluorescence **(J)** of J2341:GFP signal in the 5-day-old WT and transgenic seedlings treated with 100 mM NaCl for 24 h; Data represent means ± SD from three biological replicates. “*” *p* < 0.05; “**” *p* < 0.01; “***” *p* < 0.001; n.s. means no significant difference based on an unpaired *t*-test.

To further explore the role of *NbCIPK25* in root meristem development under salt stress, we analyzed the expression of the QC-specific gene WOX5 and the columella initial cell marker J2341 (Pi et al., 2015). The WOX5 signal was weakened by salt treatment in both WT and *NbCIPK25* transgenic lines. Unexpectedly, *NbCIPK25* overexpression did not affect the WOX5 signal. Nevertheless, the J2341:GFP signal was significantly affected upon *NbCIPK25* overexpression. For plants grown under normal conditions, J2341 showed higher expression in the columella initial cells of WT than in those of transgenic plants, as assessed by quantifying the fluorescence area, intensity, and total fluorescence derived from J2341:GFP (Figures 6A,B,E,G,H). As expected, salt stress decreased the J2341:GFP signal in both WT and transgenic seedlings. Interestingly, however, the J2341:GFP signal in transgenic plants showed a broader area of expression than in WT seedlings under salt stress (Figures 6C,D). Consistent with these results, *NbCIPK25*-overexpressing seedlings had a more extensive area of J2341:GFP expression (Figure 6F), greater fluorescence intensity (Figure 6I), and greater overall fluorescence (Figure 6J) compared with WT seedlings under salt treatment. Hence, we conclude that *NbCIPK25* overexpression, in the J2341:GFP background, maintains columella initial cells of the root apical meristem in salt-stressed plants.

### *NbCIPK25* affects the distribution of auxin in root meristem cells in salt-stressed plants

**FIGURE 7:**
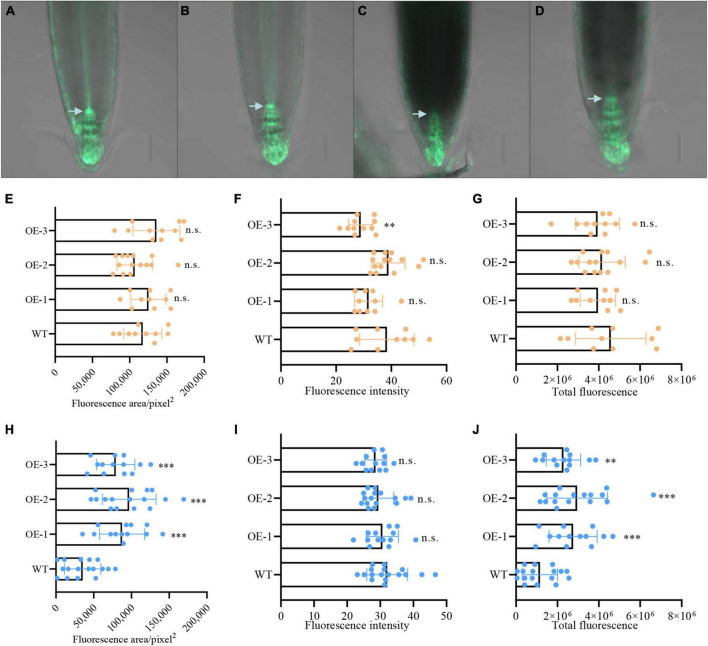
*NbCIPK25* contributes to auxin distribution at the root meristem under salt stress. **(A–D)** Green fluorescence indicates the DR5:GFP signal in WT **(A)** and transgenic seedling **(B)** treated with 0 mM NaCl for 24 h; DR5:GFP in WT **(C)** and transgenic seedling **(D)** treated with 100 mM NaCl for 24 h; scale bar: 50 μm; arrowheads indicate the QC. **(E–G)** Fluorescence area **(E)**, fluorescence intensity **(F)**, and total fluorescence **(G)** of the DR5:GFP signal in the 5-day-old WT and transgenic seedlings treated with 0 mM NaCl for 24 h. **(H–J)** Fluorescence area **(H)**, fluorescence intensity **(I)**, and total fluorescence **(J)** of the DR5:GFP signal in the 5-day-old WT and transgenic seedlings treated with 100 mM NaCl for 24 h. **(E–J)** Data represent means ± SD, three biological replicates. “**” *p* < 0.01; “***” *p* < 0.001; n.s. means no significant difference via an unpaired *t*-test.

Auxin plays an essential role in regulating the stem-cell niche and root development in response to salt stress (Liu et al., 2015; He et al., 2018; Wang et al., 2018). To study whether *NbCIPK25* expression affects auxin signaling in the root during salt treatment, the synthetic auxin-induced transcriptional reporter DR5 was introduced into *NbCIPK25* transgenic plants for subsequent analysis of the auxin spatiotemporal distribution in the root apical meristem. The DR5 signal could be observed mainly in the QC, columella initial cells, and columella cells of the root apical meristem. Under normal conditions, DR5::GFP distribution differed only negligibly at the root meristem area between WT and transgenic plants (Figures 7A,B,E); the GFP fluorescence intensity of *NbCIPK25*-overexpressing plants was lower than that of WT (Figure 7F); however, total fluorescence did not differ significantly between WT and transgenic lines grown on a medium without salt (Figure 7G). Under salt stress, DR5::GFP fluorescence was reduced in the root tips of both WT and transgenic seedlings (Figures 7A–D). However, the salt stress-induced decreases in both fluorescence area (Figure 7H) and total fluorescence (Figure 7J) in *NbCIPK25*-overexpressing seedlings were significantly less dramatic than in WT seedlings. In WT, the root meristem area marked by DR5::GFP decreased more in the QC than in columella initial cells (Figures 7A–D). These results indicate that *NbCIPK25* counteracts the reduction of auxin in the root meristem in response to salt stress.

### *NbCIPK25* mitigates H_2_O_2_ accumulation and cell damage at the root meristem of *Arabidopsis* under salt stress

**FIGURE 8:**
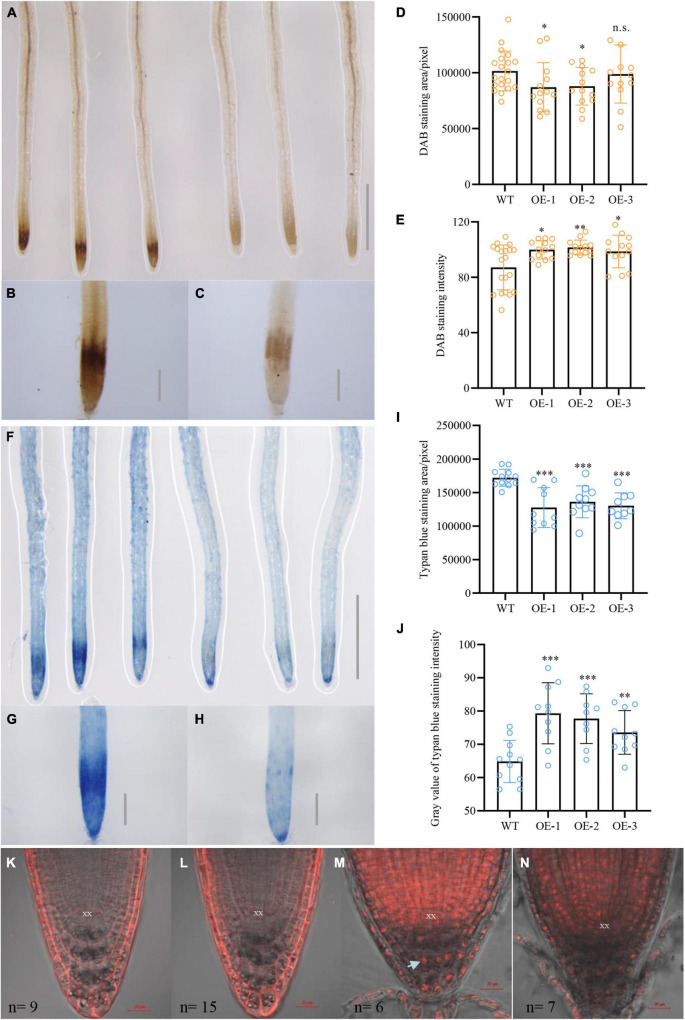
*NbCIPK25* overexpression reduces H_2_O_2_ content and cell damage caused by salt stress. **(A–C)** DAB staining for H_2_O_2_ of WT (left three roots in **A** and **B**) and transgenic seedling roots (right three roots in **A** and **C**) treated with 100 mM NaCl for 2 days, scale bar in A: 0.1 cm; scale bar in B; and C: 0.01 cm. **(D,E)** Statistical analysis of DAB staining area **(D)** and intensity **(E)** for roots in **(B)** and **(C)**, data represent the means ± SD from three biological replicates. A *t*-test was used to determine statistical significance. “*” *p* < 0.05, “**” *p* < 0.01, n.s.: not significant. **(F–H)** Trypan blue staining for cell damage in WT (left three roots in **F** and **G**) and transgenic seedling roots (right three roots in **F** and **H**) treated with 100 mM NaCl for 2 days; scale bar in f: 0.1 cm; scale bar in g and h: 0.01 cm. **(I–J)** Statistical analysis of trypan blue staining area **(I)** and gray value **(J)** for roots in **(G,H)**. **(K–N)** PI staining of the 5-day-old WT and transgenic seedlings grown for 24 h in the absence of salt (panels **K** and **I**, respectively) or in the presence of 100 mM NaCl (**M** and **N**, respectively). The arrowhead indicates a nucleus and n indicates the number of observed plants. “*” *p* < 0.05, “**” *p* < 0.01, “***” *p* < 0.001, n.s.: no significant difference as determined from a *t*-test.

Previous research demonstrated that salinity-induced ROS accumulation perturbs auxin homeostasis and its associated signaling, thereby disrupting the elongation of primary roots (Liu et al., 2016). To explore whether *NbCIPK25* overexpression leads to changes in ROS accumulation, we performed DAB staining to detect H_2_O_2_ in plant cells (Romero-Puertas et al., 2010). This revealed more intense staining in the root meristem of WT seedlings than transgenic seedlings treated with 100 mM NaCl for 2 days (Figures 8A–C), but no difference between WT and transgenic seedlings grown under normal conditions (Supplementary Figure 1A). The roots of *NbCIPK25*-overexpressing seedlings showed a significantly smaller area with intense DAB staining than WT roots (Figure 8D). Furthermore, the gray level calculated for DAB staining in transgenic seedlings was significantly higher than in WT seedlings, indicating less intensity of DAB staining in the transgenic seedlings (Figure 8E). In addition, we measured the O_2_^–^ content of salt-stressed plants, revealing no substantial difference between WT and transgenic plants (Supplementary Figure 1B). These results show that *NbCIPK25* mitigates H_2_O_2_ levels under salt stress.

According to a previous study, ROS accumulation facilitates PCD in vital meristematic tissues in the root tip to protect the QC from damage (Jin et al., 2010). Thus, to analyze whether lowered ROS levels in transgenic plants indeed result in less cellular damage in the root meristem, we determined the extent of cell death in WT and transgenic plants under 100 mM NaCl treatment using trypan blue to selectively stain dead cells and tissues (Qiao et al., 2010). For seedlings treated with 100 mM NaCl for 2 days, WT roots stained more strongly than transgenic roots (Figures 8E–H), consistent with the previously performed DAB staining (Figures 8A–C). Moreover, statistical analysis revealed a significantly larger area of trypan blue staining at the root meristem of WT plants (Figure 8I). In contrast, the gray level for *NbCIPK25*-overexpressing seedlings was much higher than WT, indicating less intense trypan blue staining and thus less cell damage in transgenic roots (Figure 8J).

The trypan blue results were mirrored by a subsequent PI staining, which colors the plasma membrane of healthy cells, yet stains the nucleus of damaged cells. Under normal growth conditions, PI staining of the membrane of root cells did not differ between WT and transgenic plants (Figures 8K,L). For plants treated with 100 mM NaCl, however, transgenic plants showed relatively less PI staining of cellular nuclei of cells at the root meristem (Figures 8M,N).

**FIGURE 9:**
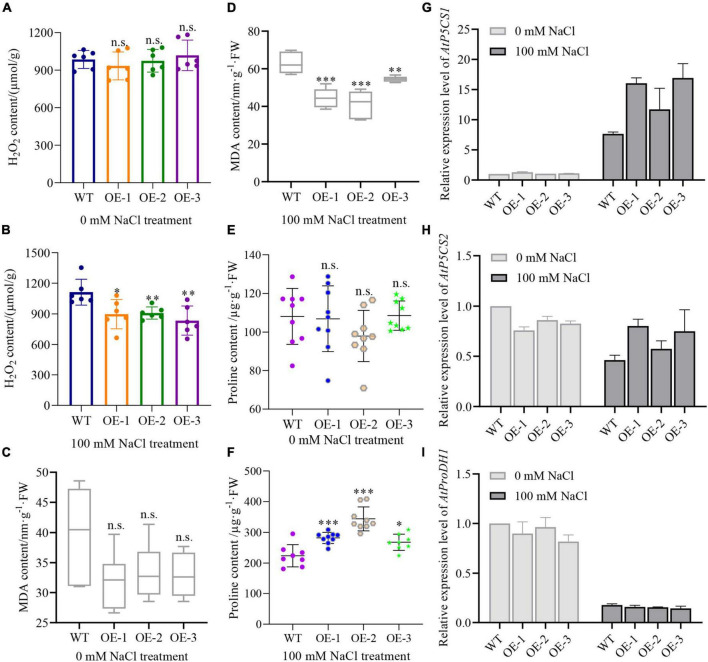
*NbCIPK25* overexpression leads to higher proline but lower H_2_O_2_ and MDA content. **(A–F)** H_2_O_2_, MDA, and proline content of the 5-day-old seedlings treated with 0 mM NaCl or 100 mM NaCl for 3 days. Data represent means ± SD, three biological replicates, a *t*-test was used to determine statistical significance, “*” *p* < 0.05, “**” *p* < 0.01, “***” *p* < 0.001, n.s.: not significant. **(G–I)** *P5CS1* **(G)**, *P5CS2* **(H)**, and *ProDH1* **(I)** transcript levels as determined by qPCR for the 5-day-old WT and *NbCIPK25* overexpressing plants treated with 0 mM NaCl or 100 mM NaCl for 2 h.

Moreover, we measured the total H_2_O_2_ and MDA content to assess the extent of lipid peroxidation (Tsikas, 2017). The results showed that there’s no significant difference in the total H_2_O_2_ content between WT and transgenic plants under normal conditions (Figure 9A). However, salt treatment induced a significantly lower H_2_O_2_ in transgenic plants than in WT plants (Figure 9B). Interestingly, MDA content in transgenic seedlings was lower than in WT under normal conditions, yet did not reach statistical significance (Figure 9C). The difference in MDA content was, however, clearly aggravated when plants were treated with salt, showing a significantly lower MDA content in transgenic plants than in WT plants (Figure 9D). These results demonstrate that *NbCIPK25* reduces H_2_O_2_ accumulation and the extent of cell damage to enhance the salt tolerance of *Arabidopsis* seedlings.

### *NbCIPK25* enhances proline accumulation under salt stress when overexpressed in *Arabidopsis*

Because proline has been identified as a crucial amino acid for protecting plant cells against ROS accumulation caused by stresses (Verslues and Sharma, 2010), to explore the effective scavenger of H_2_O_2_ in *NbCIPK25* overexpressing plants, we quantified the proline content of plants treated with 100 mM NaCl. We found that proline accumulation was not significantly different between WT and *NbCIPK25* trangenic seedlings on medium without added NaCl (Figure 9E), was substantially affected by salt treatment: the proline content of transgenic plants is significantly higher than that of WT plants after a 3-day 100 mM NaCl treatment (Figure 9F).

Consistent with these results, we found that *P5CS1*, a protein that plays a key role in proline synthesis (Verslues and Sharma, 2010), was significantly upregulated in transgenic plants in comparison to WT under salt treatment. *P5CS1* transcripts in *NbCIPK25* overexpressing seedling was 1.1-∼1.3-fold of that in WT under normal conditions (Figure 9G). Salt treatment then resulted in 7.6-fold of *P5CS1* expression in WT plants, while 11.7-∼16.9-fold in transgenic plants referenced to the gene expression level in WT under normal conditions (Figure 9G).

We then additionally tested the transcript level of *P5CS2*, which also plays a positive role in proline accumulation and the gene encoding proline dehydrogenase (ProDH1) which promotes proline catabolism. The results showed that both *P5CS2* and *ProDH1* transcripts were downregulated after salt treatment (Figures 9H,I). However, the expression level of *P5CS2* in transgenic plants was higher, while that of *ProDH1* was lower than in the WT when treated with 100 mM NaCl (Figure 9H). Together these data indicate that *NbCIPK25* mediates the expression of genes related to proline synthesis and promotes proline accumulation limiting H_2_O_2_ accumulation and lipid peroxidation, thereby protecting plant cells.

## Discussion

Numerous studies have previously been carried out concerning plant signaling pathways, that are responsive to abiotic stresses, and these studies have provided several methods for improving the adaptability and tolerance of plants grown in saline soil (Guo et al., 2001; Zhu, 2002; Sheng et al., 2009; Zhou et al., 2018; Wang et al., 2021; Zhao et al., 2022). Nevertheless, the underlying mechanisms and candidate methods mostly have originated from studies performed on glycophytes, which are sensitive to salt stress (Zhou et al., 2014; Hu et al., 2015; Zhou et al., 2018; Yin et al., 2019; Wang et al., 2021; Zhao et al., 2022). In contrast, studies of halophytes, which are better equipped to deal with salt stress than glycophytes, are relatively scarce, although these plants constitute potential resources of salt-tolerant genes (Ruifen et al., 2012; Lu et al., 2020). Here, we analyzed a salt-responsive *CIPK25* gene from the halophyte *N. billardieri* and expressed it in *Arabidopsis* for functional exploration of salt adaptability.

### A salt-induced calcium signal is necessary for CIPK functions

Our results reveal the stress mitigating role of specific genes during seedling development under salt-stress conditions. These results are reminiscent of *AtCIPK25* function in root development, where it modulates the coordination of auxin and cytokinin signaling. The *AtCIPK25* loss-of-function mutation results in shorter roots, and its overexpression leads to significantly longer roots under normal conditions (Mukesh et al., 2018). *AtCIPK25* facilitates polar auxin transport, which is thought to be one of the reasons why *AtCIPK25* causes root elongation in a normal growth environment. This research demonstrates the potential role of *CIPK25* homologs in root development (Mukesh et al., 2018).

In our present study, however, transgenic expression of halophyte-derived *NbCIPK25* in *A. thaliana* plants did not affect the length of newly grown roots under normal growth conditions but led to significantly longer roots under salt stress. This result was supported by the observation that root tips of transgenic plants were significantly longer than those of WT under salt stress. Moreover, under normal growth conditions, DR5:GFP signaling in *NbCIPK25* transgenic plants was even lower than that of WT (Figures 7F,G), yet under salt stress, DR5:GFP signaling was significantly stronger than WT (Figures 7H,J). These results underscore the clear difference in gene function during root development between the *N. billardieri CIPK25* and *A. thaliana CIPK25*.

According to the function of CIPK family genes, stress-induced calcium signaling plays an important role in activating CBL proteins, which in turn regulate the activities of CIPKs. In other words, the calcium signal acts as “a key” to activate the CBL-CIPK pathway in regulating tolerance to salinity. This is supported by a calcium-binding peptide (CBP) increasing calcium signal to enhance salt tolerance (Tsou et al., 2012). *CBP* overexpression not only induces more total Ca^2+^ and increased salt tolerance but also leads to an upregulation of *CIPK6* expression and increased root growth. This demonstrates that Ca^2+^ directly participates in signal transduction to alter gene expression and improve salt tolerance (Tsou et al., 2012). Therefore, it is not difficult to understand why *NbCIPK25* in transgenic *Arabidopsis* plants remains relatively inactive under normal growth conditions, however, becomes activated in response to salt stress. This is consistent with results from a study of chickpea *CaCIPK25*, which also has been proposed as a positive regulator of root development in response to salinity (Meena et al., 2015).

### Proline biosynthesis mediates a decrease in salt-induced H_2_O_2_ accumulation in *NbCIPK25* transgenic plants

Previous reports have shown that proline accumulation stimulates salt tolerance in plants, by acting as an osmolyte, a metal chelator, and an antioxidative defense molecule (Meena et al., 2019). Increasing proline levels as a result of the overexpression of *P5CS* genes in response to stress-induced ROS is a well-documented phenomenon in plants (Kishor et al., 1995; Munns, 2005). Knockout mutants of *P5CS* display a reduction in proline levels, resulting in ROS accumulation, which limits root elongation and enhances chlorosis and seedling lethality under salt stress (Székely et al., 2008). These conclusions indicate the ability of proline biosynthesis to scavenge free radicals caused by stresses.

In our study, we tested the expression level of critical genes that participate in proline biosynthesis to analyze whether more proline production in transgenic plants correlated with lower H_2_O_2_ levels than in WT under salt stress. In line with previously reported studies, our results display upregulation of the *P5CS1* expression level even in WT suffering from salt stress (Figure 9G), with an accompanying increase in proline accumulation (Figures 9E,F). Consistent with *P5CS1* in *NbCIPK25* transgenic plants being upregulated to a much higher level than in WT plants undergoing salt treatment (Figure 9G), transgenic plants also showed more proline accumulation (Figure 9F). On the contrary, although the expression level of *ProDH1*, a gene that regulates proline catabolism, was downregulated in both WT and transgenic plants under salt treatment, it showed a similar expression level in both lines (Figure 9I). These data indicate that the higher proline accumulation in *NbCIPK25* overexpressing plants results from more efficient synthesis, not slower catabolism of proline.

A previous study performed in mammalian cells has shown that the function of proline on ROS reduction depends on utilization of the proline biosynthetic pathway, not on proline itself, whose results revealed that cells utilize proline biosynthesis as a “vent” to prevent ROS generation due to the assimilation of extra electrons from too much buildup of NAD(P)H to influence the ability of oxidative phosphorylation (Schwörer et al., 2020). It is possible that plants use a similar mechanism to combat ROS production under stressed conditions. Summarizing, our data demonstrate that *NbCIPK25* expression leads to increased proline biosynthesis, which in turn decreases H_2_O_2_ content, helping to maintain the plant root meristem undergoing salt stress.

## Conclusion

In summary, our study identified a salt-responsive *CIPK25* gene from the halophyte *N. billardieri*. *NbCIPK25* overexpression assists the root growth of plants under salt stress through regulation of auxin distribution and root meristem development. Furthermore, overexpression of *NbCIPK25* stimulates the activity of the proline synthesis pathway leading to proline accumulation, which is the likely cause of lower H_2_O_2_ levels and cell damage in transgenic plants under salt stress. Therefore, these results clearly show that *NbCIPK25* functions to enhance plant salt tolerance, further supporting the molecular breeding of ecological or commercial plants with stronger stress tolerance.

## Data availability statement

The datasets presented in this study can be found in online repositories. The names of the repository/repositories and accession number(s) can be found in the article/Supplementary Material. The gene ID for *NbCIPK25* (MZ353017) that has been released in the NCBI database. Available at: https://www.ncbi.nlm.nih.gov/nuccore/MZ353017.1/.

## Author contributions

PW, ZH, and YLu carried out the statistical analysis. LZ and JZ prepared the *N. billardieri* seedlings. YLi and YT crossed *Arabidopsis* seedlings. LL and XW performed all the leftover experiments and arranged the manuscript. JS revised the article. TC and JC designed the experiments. All authors contributed to the manuscript revision and approved the submitted version.

## Footnotes

1
http://www.ebi.ac.uk/InterProScan/

2
http://ca.expasy.org/tools/protscale.html

3
http://www.cbs.dtu.dk/services/TMHMM/

4
http://meme-suite.org/tools/meme
