# Supplementary material for: Halophyte Nitraria billardieri CIPK25 mitigates salinity-induced cell damage by alleviating H2O2 accumulation
Source: Front Plant Sci. 2022 Aug 8;13:961651. doi: 10.3389/fpls.2022.961651 (PMC9393555; doi:10.3389/fpls.2022.961651)
Supplement: Supplementary file 1 [file Data_Sheet_1.docx]

Supplementary Material

# Supplementary Figures and Tables

**1.1 Supplementary Figures**


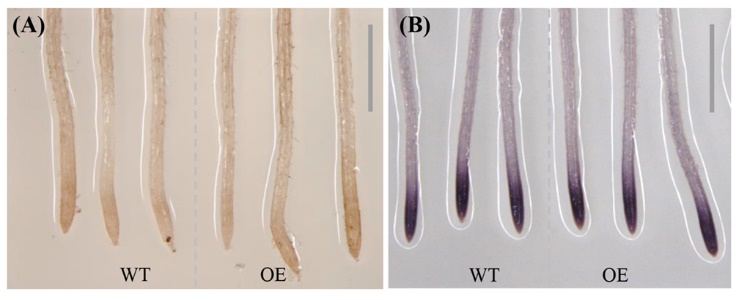


**Supplementary Figure 1. ROS detection for WT and transgenic plants.**

**(A)** DAB staining of 5-day-old WT and transgenic seedlings under normal conditions for 2 days, scale bar: 0.1 cm.

**(B)** NBT staining for O_2_^.-^ detection in 5-day-old seedlings under 100 mM NaCl treatment for 2 days, scale bar: 0.1 cm.

**1.2 Supplementary Tables**

| **Supplementary Table 1. Primers for the isolation of *NbCIPK25* fragment** | |
| --- | --- |
| **Primer name** | **Sequence (5’-3’)** |
| *CIPK25* forward primer | AACAAGGATAAAGTGAAGAAGGAAGT |
| *CIPK25* reverse primer | CCTGACAAAACTTAACATACTCCAAG |

| **Supplementary Table 2. Primers for RACE** | |
| --- | --- |
| **Primer name** | **Sequence (5’-3’)** |
| *CIPK25*:3’race primer A | CAATTCCGGCCATCATGCGTCTCCCCT |
| *CIPK25*:3’race primer B | AGGTTACAGGGGAAATTCGAGGGGAGG |
| *CIPK25*:3’race primer C | GAAGTTGCGGTGGTGGAATTCTCGAAGT |
| *CIPK25*:5’race primer A | CCTTAAAACCTCCGGCGCCACGTAAGCA |
| *CIPK25*:5’race primer B | ACTGTGTGTGGAGGAGCCCGTCGTTG |
| *CIPK25*:5’race primer C | ATCGACGGCGCTGATCAACTGCT |

| **Supplementary Table 3. Primers for complete coding region of *NbCIPK25* gene** | |
| --- | --- |
| **Primer name** | **Sequence (5’-3’)** |
| *CIPK25*:wl F | GCTCTAGAATGGCGGAGGAGGATAAAC |
| *CIPK25*:wl R | TCCCCCGGGAACTTCCACAGTCATCTCATCTC |

| **Supplementary Table 4. CIPKs from other species for conserved domain analysis** | | |
| --- | --- | --- |
| Protein name | Accession number | Species name |
| TcCIPK25 | EOY29347.1 | *Theobroma cacao* |
| VvCIPK25 | RVW54172.1 | *Vitis vinifera* |
| RcCIPK25 | XP_002515434.1 | *Ricinus communis* |
| JcCIPK25 | XP_012076439.1 | *Jatropha curcas* |
| PtCIPK25 | XP_002308703.2 | *Populus trichocarpa* |
| PaCIPK25 | TKS11827.1 | *Populus alba* |
| AtCIPK25 | AED93402.1 | *Arabidopsis thaliana* |

| **Supplementary Table 5. CIPK family proteins in Arabidopsis for phylogenic analysis** | |
| --- | --- |
| Gene name | Accession number |
| AtCIPK1 | AAG28776.1 |
| AtCIPK2 | AAF86506.1 |
| AtCIPK3 | AEC07917.1 |
| AtCIPK4 | AAG01367.1 |
| AtCIPK5 | AAF86504.2 |
| AtCIPK6 | AEE85835.1 |
| AtCIPK7 | AEE76704.1 |
| AtCIPK8 | AEE84900.1 |
| AtCIPK9 | AEE27245.1 |
| AtCIPK10 | AED97046.1 |
| AtCIPK11 | AAK16686.1 |
| AtCIPK12 | AEE84078.1 |
| AtCIPK13 | AEC08929.1 |
| AtCIPK14 | AED90397.1 |
| AtCIPK15 | AED90395.1 |
| AtCIPK16 | AAK50348.1 |
| AtCIPK17 | AAK64513.1 |
| AtCIPK18 | AAK59695.1 |
| AtCIPK19 | AAK50347.1 |
| AtCIPK20 | AED95303.1 |
| AtCIPK21 | AAK59696.1 |
| AtCIPK22 | AEC09543.1 |
| AtCIPK23 | AAK61494.1 |
| AtCIPK24 | AED93966.1 |
| AtCIPK25 | AED93402.1 |
| AtCIPK26 | AED92948.1 |

| **Supplementary Table 6. Homologous CIPK25 from other plant species for phylogenic study** | |
| --- | --- |
| Protein name | NCBI accession number or link |
| *Populus alba* CIPK25 | TKS11827.1 |
| *Populus trichocarpa* CIPK25 | XP_002308703.2 |
| *Ricinus communis* CIPK25 | XP_002515434.1 |
| *Jatropha curcas* CIPK25 | XP_012076439.1 |
| *Aquilegia coerulea* CIPK25 | PIA61311.1 |
| *Theobroma cacao* CIPK25 | EOY29347.1 |
| *Carica papaya* CIPK25 | XP_021890649.1 |
| *Vitis vinifera* CIPK25 | RVW54172.1 |
| *Linum usitatissimum* CIPK25 | Lus10007448 (link: string) |
| *Brassica oleracea* CIPK25 | XP_013596800 |
| *Arabidopsis lyrate* CIPK25 | XP_002874241.1 |
| *Boechera stricta* CIPK25 | Bostr.5763s0010.1.p (link: string) |
| *Capsella rubella* CIPK25 | XP_006289304.1 |
| *Gossypium raimondii* CIPK25 | XP_012447532.1 |
| *Manihot esculenta* CIPK25 | XP_021629121.1 |
| *Erythranthe guttata* CIPK25 | XP_012855835.1 |
| *Populus euphratica* CIPK25 | ACN76475.1 |
| *Sorghum bicolor* CIPK25 | ACQ83494.1 |
| *Malus domestica* CIPK25 | ALT16782.1 |
| *Oryza sativa* CIPK25 | ACU57073.1 |
| *Triticum aestivum* CIPK25 | AJR22383.1 |
| *Apostasia shenzhenica* CIPK25 | PKA47323.1 |
| *Colletotrichum shisoi* CIPK25 | TQN68126.1 |

| **Supplementary Table 7. Genes related to proline accumulation in Arabidopsis for real time-PCR analysis** | | | |
| --- | --- | --- | --- |
| **Gene name** | **Accession number** | **Primer name** | **Sequence (5’-3’)** |
| *NbCIPK25* | MZ353017 | *NbCIPK25* F | AAGTCGGCTGGAGATACCTT |
|  |  | *NbCIPK25* R | ACAACATTGTCACCTTGCCAT |
| *NsActin2* | AB617805 | *NsActin* F | CATCCCTCATCGGAATGGAAGC |
|  |  | *NsActin* R | GGTAGACCCACCACTAAGCACAATG |
| *AtP5CS1* | AT2G39800 | *AtP5CS1* F | GGGTCGAAGGATTACTTACAACGAG |
|  |  | *AtP5CS1* R | TTGGATGGGAATGTCCTGATGGGT |
| *AtP5CS2* | AT3G55610 | *AtP5CS2* F | CGTAGGATTTAGTGTTCCTTGAGGT |
|  |  | *AtP5CS2* R | GACATGAAGAGAAGCTGCTAAACATTTCAC |
| *AtProDH1* | AT3G30775 | *AtProDH1* F | TCTCCGACGCGCTTATGAGAAC |
|  |  | *AtProDH1* R | ATACTCTCTCTTTACGCAATCCCGGC |
| *AtUBQ10* | At4g05320.2 | *AtUBQ10* F | CCGGAAAGACCATCACCCTTG |
|  |  | *AtUBQ10* R | TGTAGTCGGCCAAAGTACGTC |
